# Avoidance of milk and dairy products after oral surgery—is such a recommendation still valid? A cross-sectional study among German and international oral and maxillofacial surgeons and dental practitioners with review of the literature

**DOI:** 10.1007/s10006-021-01017-y

**Published:** 2021-10-25

**Authors:** Schiwa Seyedi Moghaddam, Andreas Neff

**Affiliations:** 1 Dental Office Dr. Jalali Sohi, 63796 Kahl am Main, Germany; 2 Philipps University of Marburg, Biegenstraße 10, 35037 Marburg, Germany; 3 Klinik and Poliklinik für Mund-, Kiefer- and Gesichtschirurgie (Oral and Maxillofacial Surgery), Universitätsklinikum Marburg, 35033 Marburg, Germany

**Keywords:** Milk, Dairy products, Oral surgery, Wound healing

## Abstract

**Purpose:**

For prevention of wound-healing complications, patients in German-speaking countries are traditionally advised to avoid consumption of milk and dairy products after oral surgery. In the absence of national and international guidelines, this study investigates scientific evidence and compares international practice, frequency scale, and rationale behind such recommendation.

**Methods:**

Comparison of a German cross-sectional mono-center-questionnaire pilot study and a survey among international oral and maxillofacial surgeons (OMFS), specialized oral surgeons and general dentists, evaluating international practice regarding post-operative dietary and nutrition recommendations. Our literature review further assessed scientific evidence for relevant effects of probiotics, prebiotics, and/or synbiotics.

**Results:**

Among German study participants, 56% (*n* = 64/114) advise patients to avoid milk and dairy products, with 42% of OMFS (*n* = 38) and 65% (*n* = 76) of the general dentists recommending abstention (*p* = .027). In striking contrast, such recommendation could not be identified in our international survey (*n* = 143) (*t* test, *p* < .001) nor in the literature. There were significant differences between German and international study participants regarding the rationale for dietary recommendations, with dental schools and literature most frequently indicated as sources (Fisher’s exact test, *p* < .001).

**Conclusion:**

The hypothesis of a harmful effect of the consumption of milk and dairy products after dentoalveolar surgery could not be supported by evidence. The recommendation to avoid dairy products post-surgery was identified as a specific phenomenon practiced almost exclusively in German-speaking countries. Corresponding recommendations, most probably based on a now irrelevant risk of contracting tuberculosis from milk products, can at present no longer be substantiated.

## Introduction

Nearly 3.5 billion people worldwide are affected by oral diseases [1]. According to statistics, *dolor post extractionem* occurs in 3 to 4% of all tooth extractions [2]. In order to avoid serious complications, patients are routinely advised to abstain from nicotine, alcohol, and caffeine after dental surgery, among others.

In addition, patients in German speaking countries are traditionally advised to avoid milk and dairy products after dentoalveolar surgery. This recommendation can be found in postoperative recommendations for patients as issued by health insurance companies, on numerous internet pages of the German lay press and in internet forums [3]. A Google search for the German search terms “dairy products” and “tooth” (“Milchprodukte“ & ”Zahn“) returned 354,000 entries; a search for the German for “milk” and “implant” (“Milch“ & “Implantat“) as many as 820,000 entries, the great majority of which on the subject of abstention from milk and dairy products after oral surgery [4, 5]. However, no guidelines or evidence for a possible causal link with intraoral infections of wounds exist for the consumption of milk and dairy products following dentoalveolar surgery, neither in Germany, e.g., from the Association of Scientific Medical Societies in Germany (Wissenschaftliche Medizinische Fachgesellschaften, AWMF) nor worldwide, e.g., from the American Dental Association (ADA) or the National Institute for Health and Care Excellence (NICE). We investigated the recommendations as made by dentists and OMFS in Germany by way of a pilot study based on a representative collective of general dentists in private practice and specialized OMFS, with the result that not only more than half of German general therapists; also, over 40% of specialists for oral surgery and OMFS advised to abstain from consumption of milk and dairy products after dentoalveolar surgical procedures [6]. The study also revealed a major inhomogeneity of the reasons given for such a recommendation [6] (see Table 1).

**Table 1:** Arguments in favor of abstention from milk and dairy products after dentoalveolar surgery and their frequency, compared between German and international sample

Arguments in favor of abstention from milk and dairy products after dental procedure	Frequency Germany	Frequency International
a. Milk and dairy products increase risk of infection	38.6% **^a,b^	19.8% ***^a,b^
b. Lactic acid bacteria may adversely affect wound healing	30.7% **^a,^ *^b^	15.8% ***^a,b,^ **^c^
c. Possible adverse effect on development of coagulum, coagulum surface may disintegrate	23.7% *^a^	12.2% ***^a,^ **^b^
d. Possible reduced efficiency of antibiotics	5.3% **^b^	2.7%
e. Dairy products my cause tuberculosis infection (specifically unpasteurized raw milk products)	1.8%	0.9%
f. milk may react with resorbable suture (adhesion, mucous congestion)	1.8%	0.9% *^c^

*Correlation is significant at *p* < 0.05 level (Fisher's exact test)

**Correlation is significant at *p* < 0.01level (Fisher's exact test)

***Correlation is significant at *p* < 0.001 level (Fisher's exact test)

^a^Correlation oral surgeons/OMFS ^b^ Correlation dentists/OMFS ^c^ Correlation dentists/oral surgeons

We were wondering how relevant recommendations are in the rest of the world compared to the above. The study in hand aims for clarification of the following questions: (a) How do recommendations of German dentists and OMFS differ from recommendations worldwide? (b) Which pathophysiological reason supports the recommendation to abstain from dairy products? (c) Does a review find evidence for such a recommendation?

## Material and methods

### Literature review

Electronic and manual literature searches were conducted, and studies published until January 2021 were considered. A PubMed search to retrieve the pertinent literature published in English was made, using the following search strings: “milk products“ AND ”oral surgery“, ”dairy products“ AND “oral surgery“, “dairy“ AND “wound healing“, “dairy“ AND “wound infection“, “milk“ AND “wound infection“, “probiotics“ AND “wound infection“, “dry socket“ AND “dairy“, “dry socket“ AND “milk“, “probiotics” AND “wound healing.” Study selection, validation of eligibility, and quality assessment were performed by two independent reviewers. Disagreement was resolved on the basis of discussion.

In addition to a search for pertinent literature in German through EMBASE, a manual search of the historic collection of the library at the Philipps-Universität Marburg and the German National Library (Deutsche Nationalbibliothek) was conducted.

For our probiotics review we included in vitro studies, animal studies and clinical studies in which wound healing and wound infection under therapy with probiotics was examined. We included all RCT studies on all types of probiotic, prebiotic, or synbiotic treatments in humans, aimed to examine postoperative complications after surgery.

Due to the small number of relevant studies, all available studies covering the effects of milk and dairy products (with the exception of probiotics) on wounds and saliva composition up to and including January 2021 were included. We excluded studies that did not inform about sample sizes and the mean results. Also, we excluded duplicate studies and experiments that include repeated data.

### German pilot study and international survey

Concerning recommendations by German dentists on consumption of dairy products after dentoalveolar surgery, the data had been compiled during our preceding pilot study, with a total of 114 participating dentists and OMFS which has been already published in German before [6].

To investigate relevant recommendations in other countries, in Europe, the members of the European Society of TMJ Surgeons (ESTMJS) and the Italian Academy of Osseointegration (IAO) were contacted in writing; so, there were an additional 143 active surgeons worldwide from the areas of OMFS, dentoalveolar surgery, and periodontology, with a request for information both on the general received scientific consensus in the relevant country on recommendations on the consumption of milk and dairy products following dentoalveolar surgery and on own individual practice.

The power of the questionnaire was calculated using PASS 14.0.9 statistics software. It could be established that from a minimum number of 88 eligible data sets and an equivalence distance of 0.05 the power was 0.95. Significant differences between the groups “disapprove of abstinence” and “support recommendation for abstinence from milk and dairy products after oral surgery” were therefore detected (*t* test). Significance level was *p* < 0.05.

## Results

### Literature review

A total of 380 abstracts were identified through electronic search, after removal of duplicates. Of these, 49 articles were assessed for full-text evaluation, resulting in a final selection of 36 articles for qualitative assessment. The German-language literature search in the historic collection of the Philipps-Universität Marburg produced two volumes which matched our inclusion criteria.

Probiotics were shown to promote wound healing in in vitro studies and various human and animal models by increasing collagen concentration, granulation tissue, and angiogenesis [7–37]. Please refer to Tables 2, 3, and 4 for treatment indications and used probiotics. In one study, the mean outcome demonstrated no significant difference in wound healing after third molar extraction between the probiotic group and the control group [39].

**Table 2:** Characteristics of 17 animal model studies of the effect of probiotics on wound healing

First author, year	Study type	Probiotic	Findings (effect of probiotics on wound)
Valdez, 2005 [7]	Animal model study and in vitro study	*L. plantarum*	Enhanced wound healing
Brachkova, 2011 [9]	Animal model study	*L. plantarum*	Reduction of *Pseudomonas aeruginosa* in wound
Nasrabadi, 2011 [10]	Animal model study	*L. brevis* and *L. plantarum*	Decreased inflammation and improved wound healing
Jones, 2012 [11]	Animal model study	*L. fermentum*	Enhanced wound healing
Poutahidis, 2013 [12]	Animal model study	*L. reuteri*	Enhanced wound healing
Partlow, 2016 [14]	Animal model study	*S. bouladrii*	Unimproved wound healing, unchanged microbiome
Argenta, 2016 [15]	Animal model study	*L. plantarum*	Decreased mortality and production of inflammatory markers (TNF-α, IL 6 and 10), less septicemic accumulation in organs
Satish, 2017 [16]	Animal model study	*L. plantarum*	Reduced severity and length of infection and collagen concentration (less scarring)
Oryan, 2018 [17]	Animal model study	*S. cerevisiae*	Enhanced burn wound healing
Ong, 2019 [18]	Animal model study	*L. plantarum*	Enhanced wound healing, suppressed *Staphylococcus aureus* infection at wound
Tagliari, 2019 [19]	Animal model study	*L. paracasei*, *B. lactis*, *L. rhamnosus*, *L. acidophilus*	Enhanced wound healing, increased collagen production
Khodaii, 2019 [20]	Animal model study	*L. reuteri*	Enhanced collagen production, accelerated wound healing
Han, 2019 [21]	Animal model study	*L. reuteri*	Promoted wound healing through activation of GMSCs
Han, 2020 [26]	Animal model study	*L. reuteri*	Inhibited inflammation and enhanced wound healing through promoting MSCs
Mohtashami, 2020 [23]	Animal model study	*L. bulgaricus*, *L. plantarum*	Enhanced wound healing
Ong, 2020 [24]	Animal model study	*L. plantarum*	Suppressed *Staphylococcus aureus*, and thereby improved wound healing
Moreira, 2021 [25]	Animal model study	*L. johnsonii*, *L. paracasei*, and *L. rhamnosus*	Promoted skin wound closure and decreased scar formation and inflammation

L., *Lactobacillus*; S., *Saccharomyces*; B, *Bifidobacterium*; GMSC, gingival mesenchymal stem cells; MSC, mesenchymal stem cell

**Table 3:** Characteristics of 13 studies in humans with probiotics on wound healing

First author, year	Study type	Patients: probiotic/control group	Probiotic	Findings: Effect of probiotics on wound
Rayes, 2002 [27]	Prospective	30/30	*L. plantarum*	Incidence of infections significantly lower
Kanazawa, 2005 [28]	RCT	21/23	*L. casei*, *B. breve*	Decreased quantity of pathogens in symbiotic group, thereby less surgical-site infections
Sugawara, 2006 [29]	RCT	40/41	*L. casei*, *B. breve*	Incidence of postoperative infectious complications lower
Rayes, 2007 [30]	RCT	40/40	*P. pentosaceus*, *L. mesenteroides*, *L. paracasei* subsp. *paracasei*, *L. plantarum*	Incidence of postoperative bacterial infections significantly lower with *Lactobacilli*
Peral, 2009 [31]	RCT	38/42	*L. plantarum*	Non-infected 3rd-degree burn: no effect Infected 2nd-degree burn: suppressed pathogens, enhanced wound healing Infected 3rd-degree burn: improved wound healing
Peral, 2010 [32]	Pilot study	34/0	*L. plantarum*	Decreased wound bacterial load and stimulated wound healing
Liu, 2011 [38]	RCT	50/50	*L. plantarum, L. acidophilus*, *B. longum*	Enhanced transepithelial resistance, reduced blood enteropathogenic bacteria, fewer surgical site infections
Usami, 2011 [33]	RCT	32/29	*L. casei*, *B. breve*	No infectious complications
Zhang, 2012 [34]	RCT	30/30	*B. longum*, *L. acidophilus*	Decreased postoperative occurrence of infectious complications
Kotzampassi, 2015 [35]	RCT	84/80	*L. acidophilus*, *L. plantarum*, *B. lactis*, *S. boulardii*	Decrease in incidence of postoperative pneumonia and of surgical site infections
El Ghazely, 2016 [36]	RCT	20/20	*L. fermentum*, *L. delbrueckii*	Reduction in incidence of infections, wound healing enhanced
Komatsu, 2016 [37]	RCT	168/194	*L. casei*, *B. breve*	Synbiotics suppress increase in potentially pathogenic species, less surgical site infections (not significant)
Wälivaara, 2019 [39]	RCT	32/32	*L. reuteri*	No significant impact of probiotic application on wound healing after third molar extraction

L., *Lactobacillus*; S., *Saccharomyces*; B, *Bifidobacterium*; P., *Pediococcus*

**Table 4:** Characteristics of 6 trials with dairy products on wound healing

First author, year	Study type	Dairy product	Sample size Treatment/control group	Findings
Rodrigues, 2005 [8]	Animal model study (rats)	Kefir	5 groups of 5 members	Kefir enhanced wound healing
Badr, 2012 [40]	Animal model study (diabetic mice)	Whey protein	40/20	Whey protein enhanced wound healing in diabetic mice
Schutt, 2014 [41]	Cross-over study (clinical diagnosis)	Ice cream	6/6	Consumption of dairy products had no influence on microbiome in oral cavity
Rouabhia, 2017 [42]	In vitro study	Yogurt		Yogurt enhanced gingival epithelial cell growth and keratin K5 production
Hemmati, 2018 [13]	Animal model study (rabbit)	Low-fat milk ointment	6 groups of 6 members	Low-fat milk ointment enhanced wound healing rate
Oryan, 2018 [17]	Animal model study (burn wound from rats)	Kefir	12	Improvement of angiogenesis and proliferation of fibroblasts

### German pilot study and international survey

For our German pilot study [6], a total of 150 dentists (*n* = 103), specialist dentists for oral surgery (*n* = 30), and OMFS (*n* = 17) were contacted in writing. The overall response rate for all questionnaires in the German pilot study was 76% (*n* = 114, of which 76 dentists, 25 specialist dentists for oral surgery and 13 OMFS). For our international survey, we contacted 143 active specialist surgeons in the areas of OMFS (*n* = 61), dentoalveolar surgery (*n* = 6), and periodontology (*n* = 76) in writing. Here, the overall response rate was 75.5% (*n* = 108, 45 of which OMFS, 6 specialist dentists for oral surgery and 57 periodontists) (Table 5). For the nationalities of the participants in the international survey by questionnaire and its result, please refer to Table 6. Our international survey revealed that only in the German-speaking areas and Flanders dentists continue to recommend abstention from consumption of dairy products after dentoalveolar surgery (Table 6). Globally, a total of 95.3% of study participants stated they did not make any recommendation to abstain from consumption of milk and dairy products after dentoalveolar surgery. Those 4.7% surveyed who advised so were all from German-speaking and Flemish-speaking geographies (Germany, Austria, Switzerland, and Belgium). Actually, in Italy, India, Thailand, and the USA, some surgeons explicitly recommend the consumption of dairy products, e.g., milk shakes, ice cream, or yoghurt.

**Table 5:** Response rate and study participants by specialization, in Germany and international

Specialization	Contacted Germany	Response rate Germany	Response rate Germany	Contacted International	Response rate International	Response rate International
OMFS	17	13	76.5%	61	45	73.8%
Oral surgeon	30	25	83.3%	6	6	100.0%
Dentist/periodontist	103	76	73.8%	76	57	75.0%
Total	150	114	76.0%	143	108	75.5%

**Table 6:** Guidelines or recommendations regarding milk and dairy products after oral surgery and comments by country

Country	Guidelines or recommendations	Comments
Argentina	Yes/no	Avoidance only in the older generation of surgeons because of resorbable suture materials used formerly such as catgut and because of increasing bacteria level and metabolism
Austria	Yes	
Belgium (Flemish)	Yes	Whenever patient is unable to clean the mouth properly: avoidance of milk-based products in the first week to avoid fermentation and bad oral odor
Belgium (Walloon)	No	No restrictions. On the contrary: milkshakes with eggs and sugar and cream (calories and proteins) recommended
Brazil	No	
Chile	No	The protocols issued by universities and Health Ministry do not avoid milk or dairy. Also, the Universidad Austral in the city of Valdivia (area with greatest German influence) does not make recommendations to avoid milk or dairy after oral surgery
China	No	The older generation recommended to avoid food products which are infested with bacteria rapidly, i.e., within a few hours or with change in quality difficult to detect. With the wide availability of frozen food and refrigerators, foods can be stored for a much longer time without degenerating
Denmark	No	
England	No	
Estonia	No	
France	No	
Germany	Yes	
India	No	Consumption of milk and dairy products recommended after all minor oral surgery procedures. A usual practice is asking the patient to start having ice creams around 30 min to 1 h post any minor surgical procedure
Iran	No	
Israel	No	
Italy	No	Some surgeons suggest a liquid or soft and creamy diet (yoghurt or milk shakes in particular)
Japan	No	
Lithuania	No	
Malaysia	No	
Netherlands	No	
Norway	No	
Portugal	No	
Slovenia	No	
Slovakia	No	
Sweden	No	
Switzerland	Yes	
Spain	No	
Thailand	No	Patients who cannot chew well after receiving oral surgery are recommended to eat ice cream or yoghurt.
Yemen	No	
USA	No	

In contrary, in Germany, the result was almost diametrically opposed to the international survey. Here, 56.1% of the sample stated that they advised against consumption of dairy products after surgical procedures in the oral cavity—especially when patients inquire accordingly (see Table 7). While statistically there is no unequivocal consensus or even a clearly prevalent opinion among the German survey, a comparison between the international survey and the German survey nevertheless shows a highly significant difference.

**Table 7:** Recommended behavior after dentoalveolar surgery in relation to milk and dairy products by dentistry specialty in Germany and International

Region	Dentistry specialty	Recommendation to abstain from milk/dairy products	No recommendation to abstain from milk/dairy products
Germany	OMFS (*n* = 13)	30.8% (*n* = 4)*^a^	69.2% (*n* = 9)
Germany	Oral surgeon (*n* = 25)	48.0% (*n* = 12)	52.0% (*n* = 13)
Germany	OMFS/oral surgeon (*n* = 38)	42.1% (*n* = 16)*^b^	57.9% (*n* = 22)
Germany	General dentist (*n* = 76)	64.9% (*n* = 49)*^a,b^	35.1% (*n* = 27)
Germany	Total (*n* = 114)	56.1% (*n* = 64)**^c^	43.9% (*n* = 50)
International	Total (*n* = 108)	4.6% (*n* = 5)**^c^	95.4% (*n* = 103)

*Correlation is significant at *p* < 0.05 level (Fisher’s exact test)

**Correlation is significant at *p* < 0.01 level (Fisher’s exact test)

^a^Correlation dentists/OMFS ^b^ Correlation oral surgeons and OMFS/dentists ^c^ Correlation total international/total Germany only

While in the international survey, recommendations are highly uniform and independent from the specialization of the OMFS, oral surgeon, or periodontist (i.e., no recommendation to abstain from dairy products), in Germany, the relevant recommendation was shown to be contingent on if there had been a completion of advanced surgical training. On inquiry by the patient, 64.9% of German general dentists recommended to abstain from dairy products, while a mere 42.1% of German OMFS and specialized dentists for oral surgery do so (Table 7), evidencing a significant difference between these two groups in the German collective (*p* = 0.027). At the same time, however, there was no significant difference in recommendations made between German OMFS and specialized dentists for oral surgery (Table 7). Table 8 shows possible sources of information which may motivate a recommendation to abstain from dairy products after surgical procedures in the oral cavity. Table 1 lists the reasons stated by participants in the German pilot study [6] and the international survey and possible arguments in favor of abstention from milk and dairy products after dentoalveolar surgery.

**Table 8:** Source of information stated by dentists, specialist dentists for oral surgery and OMFS regarding consumption of milk and dairy products after dentoalveolar surgery in Germany and international (multiple responses allowed) [6]

Source of Information	Dentists (international + Germany) (*n* = 133)	Specialist dentists for oral surgery (international + Germany) (*n* = 31)	OMFS (international + Germany) (*n* = 58)	Total (international + Germany) (*n* = 222)	Total (Germany only) (*n* = 114)
Teaching at University	29.3% (*n* = 39) ***^a^	41.9% (*n* = 13) ***^b^	0% (*n* = 0) ***^b^	23.4% (*n* = 52) ***^c^	45.6% (*n* = 52) ***^c^
Recommendations by other dentists - *Lactobacilli* could affect wound healing	12.8% (*n* = 17) *^a^	22.6% (*n* = 7) **^b^ *^a^	1.7% (*n* = 1) **^b^	11.3%* (*n* = 25) *^c^	21.1%* (*n* = 24) *^c^
Personal experience - To avoid fermentation and bad oral odor - Dairy products could increase the bacteria level and metabolism - (old) resorbable suture materials could resorb faster	12.0% (*n* = 16)	38.7% (*n* = 12) **^b^	8.6% (*n* = 5) **^b^	14.9% (*n* = 33)	25.4% (*n* = 29)
Specialist literature	6.8% (*n* = 9) **^d^	25.8% (*n* = 8) ***^b^ **^d^	0 = % (*n* = 0) ***^b^	7.7% (*n* = 17) **^c^	18.4% (*n* = 21) **^c^
Not specified	42.9% (*n* = 57)	19.4% (*n* = 6)	70.7% (*n* = 41)	46.8% (*n* = 104)	0% (*n* = 0)

*Correlation is significant at *p* < 0.05 level (Fisher’s exact test)

**Correlation is significant at *p* < 0.01 level (Fisher’s exact test)

***Correlation is significant at *p* < 0.001 level (Fisher’s exact test)

## Discussion

Contrary to the received opinion well established among German dentists and OMFS [6], no evidence can be found in the pertinent literature supporting adverse effects of consumption of milk and dairy products after dentoalveolar surgery.

According to the findings of our survey, in the majority of countries, avoidance of dairy products is not recommended after dentoalveolar surgery (see Table 6), while the majority of the international surveys adopt a neutral position. In countries such as Italy, India, Thailand, and the USA, surgeons actually make recommendation in favor of consumption of dairy products like milkshakes, ice cream, or yoghurt after dentoalveolar surgery, due to their satiating and cooling effects on the surgical site. A common practice is asking the patient to start having ice cream around 30 min to 1 h post any minor surgical procedure.

Diametrically opposed the situation in the German-speaking and related regions (e.g., Flanders in Belgium): in our German survey, a slight majority (56.1%) was reported to advise their patients against consumption of milk and dairy products [6], with a significant difference between dentists and OMFS in Germany (*p* = 0.027) (see Table 7), of which those with a focus on surgical procedures attributed at least a minor positive effect on wound healing to milk and dairy products [6]. Compared to the international collective, there was a striking difference regarding the sources of information given by OMFS and specialist dentists for oral surgery (Table 8). Oral surgeons stated lectures at university, specialist literature, personal experience, and recommendations from other dentists as sources of information significantly more frequently. We have been unable, however, to identify the exact specific original source of the recommendation to abstain from dairy products as traditionally made at many German universities. At the time of the First World War, the recommended diet [43] for patients who had sustained injuries to the jaw was liquids, specifically soup, milk in any form, dishes with eggs, and a variety of meat extracts [43]. Textbooks from the time of the Second World War still recommended a diet containing eggs, milk, and cocoa for same patients [44]. Therefore, the current recommendation was only propagated after the World War II.

Regarding the reasons given as listed in Table 1, the theory of an interaction between milk and antibiotics may apply for some classes of agents, e.g., tetracycline and quinolone (neither an antibiotic of preferred choice in dentistry). Milk and dairy products may indeed reduce bioavailability of tetracyclines by up to 80% [45]. The most frequently used antibiotics in dentistry, however, such as amoxicillin, propicillin, phenoxlmethyl penicillin, and clindamycin, and less frequently used cefaclor, cefuroxime, cefpodoxime, erythromycin, roxithromycin, clarithromycin, azithromycin, nitroimidazole, and metronidazole [46], do not interact with milk and dairy products. It is well known that milk is a good culture medium for certain bacterial colonies. As it is rich in numerous amino acids and minerals and due to the lactose contained inside, it may promote growth of bacteria, including pathogenic bacteria in the oral cavity, with adverse effect on wounds. And while lactobacilli are indeed required for the production of dairy products, such as yoghurt and cheese, exclusively such thermophile lactic acid bacteria are utilized here which are not pathogenic.

It is further argued that clotting may be inhibited by consumption of dairy products, with a risk of resulting dry socket and *dolor post extractionem*. Fibrinolytic plasminogen, which is also been found in milk, is believed to be responsible for the inhibition of clotting. The determining factor for the evaluation of the effect of plasminogen on coagulum development is concentration in milk (0.3–2.5 μg/ml) compared to serum concentration (200 μg/ml) [47]. The plasmin concentration in blood is 100–1000 times higher than in milk. The conclusion may be that the effect of milk on blood coagulation is not relevant and negligible in comparison to the concentration of plasminogen in the coagulum itself.

Some practitioners argue that milk and dairy products will react with resorbable sutures. In many resorbable suture materials, the degradation process is of a hydrolytic nature. In order for, e.g., tensile strength of suture material to be affected by dairy products, pH would need to be affected for an extended period of time. Absorbable suture materials are relatively more sensitive to the pH value than non-resorbable materials. The degradation of synthetic resorbable sutures, however, is more pronounced in an alkaline milieu in general rather than at physiological or acidic pH range [48]. Some opponents believe in a historical basis of the recommendation to avoid dairy products after oral surgery in an empirically elevated risk of contracting tuberculosis after consumption of dairy products in the past—obsolete today. Tuberculosis is a bacterial infectious disease, caused by the obligate pathogenic *Mycobacterium tuberculosis* complex, *M. tuberculosis*, *M. bovis*, and *M. caprae*. The latter two are zoonoses, communicable from infected animals to humans. A tuberculosis infection in humans most frequently develops into pulmonary tuberculosis and is currently the most widespread lethal bacterial infectious disease, with 10 Million individuals developing tuberculosis annually. According to the World Health Organization (WHO), there were about 1.4 million deaths due to tuberculosis worldwide in 2019, with Bangladesh, China, India, Indonesia, Nigeria, Pakistan, the Philippines, and South Africa accounting for approx. half of all known cases of tuberculosis [49]. A variety of tuberculosis is *M. bovis* which is transmitted through consumption of unpasteurized milk. Those affected by cervical tuberculous lymphadenitis, also called scrofula (historically known as The King’s Evil) [50], will exhibit cervical gland tumors and abscesses and lesions of the skin in the area of the affected lymph nodes. According to the WHO, extrapulmonary tuberculosis was diagnosed in 15% of the 6.3 million cases in Europe in 2016. The majority of cases of tuberculous cervical lymphadenopathy, however, occur in developing countries. In Europe, infections with Mycobacteria mainly affect immigrants and immunocompromised individuals. Women are relatively more frequently affected by scrofulosis, men by pulmonary tuberculosis [51]. The disease may recur and develop into a chronic, progressive form, *Lupus vulgaris* (*Tuberculosis luposa* or ulcerative *Lupus faciei*). The belief that raw milk may incur the risk of infection with tuberculosis was still well founded at the beginning of the twentieth century; in 1901, as many as 80% of cattle in Germany tested positive for tuberculin [52]. However, since 1997, the cattle population in Germany has been officially declared free from tuberculosis, as 99.9% of cattle are not affected [53]. Currently, the majority of cases of bovine tuberculosis occur in countries such as Ethiopia, India, and China, which also frequently report cases of tuberculosis in humans [53–57]. Why no recommendation is made in these countries to abstain from consumption of dairy products after dentoalveolar surgery yet needs to be established. In Germany, tuberculosis is a notifiable disease and affected cattle populations are required by law to be culled. Milk undergoes heat treatment (pasteurization/ultra-high temperature treatment), destroying all pathogenic germs, including Mycobacteria in the milk, and a danger of infection through consumption of milk after dentoalveolar surgery no longer exists [47, 58]. According to the Centers for Disease Control and Prevention (CDC), the risk of infection through contaminated raw milk is 150 times greater than by consuming pasteurized milk. Especially infants, pregnant women and people with weakened immune systems are in danger of illnesses and hospitalization. These pathogens are most often *Brucella*, *Campylobacter*, *Cryptosporidium*, *E. coli*, *Listeria*, *Salmonella*, and mastitic streptococci [59].

Some dentists advise against dairy products because of contained bacterial cultures. Live probiotic cultures are part of fermented dairy products and are intended to have health benefits when consumed. Those microorganisms also occur naturally in lactic acid products such as yogurt, kefir, or buttermilk. That is why we have made a literature review on probiotics to find out if there are any possible wound healing disorders or wound infections after use of probiotics. Our review also encompasses studies into the effect of probiotics and synbiotics on a variety of skin wounds including infected and non-infected wounds, diabetic wounds, and burn injury models. The studies, in vitro and in vivo, all arrived at the identical result and conclusion that probiotics and synbiotics are able to inhibit those wound pathogens which cause surgical site infections [60] and thus will sometimes be able to reduce required hospitalization and quantity of antibiotics [27, 29, 30, 34]. This may also help prevent development of multidrug resistant bacteria. Moreover, the use of probiotics helped to reduce inflammatory markers [15, 22, 61]. These results of our literature search agreed with the results of other systematic reviews into the influence of probiotics, prebiotics, and/or synbiotics [42, 62–64]. As our research question focuses on the effect of dairy products in the oral cavity, the caveat would be that the available literature mainly deals with the effect of probiotics on skin wounds. In an additional in vitro study into the post-wound interactions between gingival epithelial cells and drinkable yogurt, the authors observed a positive effect on oral wound healing. This was explained by an increase in various keratins and in β-defensin-2 production [65]. These keratins are important for the development of epidermal structures [66]. β-defensin stimulates cell migration and angiogenesis at wound sites and promotes wound closure [38, 65].

There are several limitations to this study. While the German survey may well be considered representative, the international survey so far constitutes a mere random sample of individuals. Even though they are generally based at universities, the results are not representative or robust for worldwide validity. They may, however, well constitute evidence of a trend. Further national surveys may be able to offer evidence-based validity.

As our research question investigates the effect of dairy products in the oral cavity, our review of probiotics was included with the reservation that it mainly covers skin wounds. The majority of the in vivo studies reviewed were conducted in countries in Asia and Europe. They may therefore not generally applicable for other populations.

## Conclusion

Available data indicate that the German-speaking areas differ significantly in their dietary recommendations after dentoalveolar surgery. The recommendation to abstain from milk and dairy products, as made by over 50% of dentists surveyed in the German pilot study, is neither evidence-based nor can its origin or scientific basis be established. Most plausible seems the empirical experience with historical background (scrofula), which today can safely be considered as no longer relevant due to pasteurization of milk. Taking on board the fact that the incidence of tuberculosis is on the rise again, and so are antibiotic-resistant strains, a possible transoral access of tuberculosis should not be taken lightly or disregarded.

The validity of the routine recommendation to abstain from consumption of milk and dairy products after dentoalveolar surgery, a widespread practice in Germany today, nevertheless should be challenged and re-examined.
